# The effect of pterygium on front and back corneal astigmatism and aberrations in natural-light and low-light conditions

**DOI:** 10.1186/s12886-023-03270-z

**Published:** 2024-01-04

**Authors:** Weiwei Xu, Xia Li

**Affiliations:** 1 Shanghai Aier Eye Hospital, NO. 83 Wuzhong Road, Xuhui District, Shanghai, 200031 China; 2 Shanghai Aier Eye Institute, PR, NO. 83 Wuzhong Road, Xuhui District, Shanghai, 200031 China

**Keywords:** Pterygium, Aberration, Front, Back, Low-light

## Abstract

**Background:**

To investigate the effect of different sizes of pterygium on the front and back corneal topography, refractive changes and aberrations in natural-light and low-light conditions.

**Methods:**

Sixty subjects with unilateral primary nasal pterygium were enrolled in this study. All the patients’ uncorrected, best spectacle-corrected visual acuity, corneal topographic aberration data in 3 mm and 7 mm areas were collected. The pterygium size was evaluated by the slit-lamp photography and Sirius Scheimpflug Analyzer.

**Results:**

The front topographic astigmatism values, corneal total aberrations, and higher-order aberrations in 3 mm and 7 mm areas were higher in the pterygium group than those in the control group. The pterygium horizontal length and thickness were moderately to strongly correlated with astigmatism and RMS of aberrations, while pterygium vertical length showed no or just mild correlation with the corneal astigmatism and aberrations. Compared to the readings in 3 mm area, the front and back corneal astigmatism and aberrations were larger in 7 mm area.

**Conclusions:**

Pterygium led to visual impairment by inducing astigmatism and aberrations. In low-light condition, the visual function worsened due to increased corneal astigmatism values and aberrations.

**Supplementary Information:**

The online version contains supplementary material available at 10.1186/s12886-023-03270-z.

## Background

Pterygium is a kind of wing-shaped neoformation characterized by abnormal conjunctival overgrowth onto the cornea, which influences the structure and function of the ocular surface [1]. Pterygium can impact the corneal refractive status including astigmatism, keratometry, and topography [2]. Traditional corneal topography assesses total corneal power from front surface indices, omitting direct back corneal measurement, and potentially introducing error into total corneal power and astigmatism evaluation [3–7].

Visual performance and optical quality correlated with wavefront aberrations, [8] better understanding pterygium’s impact requires evaluating corneal aberration changes caused caused by pterygium. Few studies studying have researched pterygium’s effect on back corneal indices and corneal aberrations [9–11].

To evaluate pterygium’s impact on visual function, ambient illumination must be studied. Lower ambient illumination yields larger pupil diameters than natural-light condition. In low-light conditions, larger corneal zones should be considered for evaluating astigmatism and aberrations. No data exists on pterygium-induced corneal astigmatism and aberration changes in low-light conditions.

The Sirius Scheimpflug Analyzer (CSO, Costruzione Strumenti Oftalmici, Florence, Italy) is a new Scheimpflug imaging device that measures full corneal pachymetry and anterior chamber depth (ACD), combining a single rotating Scheimpflug camera and a Placido-disk corneal topographer [12–14]. It provides accurate measurements of curvature and astigmatism at front and back corneal surfaces [15]. The sirius Scheimpflug Analyzer can also evaluate corneal total and higher-order aberrations (HOAs) accurately [15]. Moreover, it provides clinicians with data in different areas. In this study, the Sirius Scheimpflug Analyzer was used to evaluate changes in corneal indices and aberration caused by pterygium in 3 mm and 7 mm areas, simulating natural-light and low-light conditions.

## Methods

This is a retrospective control study. The aim of this study was to investigate the effect of different sizes of pterygium on front and back corneal topography, refractive changes and aberrations in natural-light and low-light conditions.

### Study population

The study included 60 subjects diagnosed with primary unilateral pterygium from January 2021 to August 2021.The eye without pterygium of the same subject was considered as control. Exclusion criteria included bilateral pterygium, recurrent pterygium, or pseudopterygium; any other ocular diseases other than pterygium; history of ocular surgeries. Demographics data were recorded.

### Ophthalmologic examination and pterygium sizes measurement

Uncorrected visual acuity (UCVA) and best-spectacle-corrected visual acuity were measured using a Snellen eye chart at 5 m. The Snellen fraction was converted to approximate ETDRS (approxETDRS) letter scores, for statistical manipulations using the strategy proposed by Ninel Z [16].

Each subject underwent comprehensive ophthalmic examination. Slit-lamp photography (Topcon SL-D Digital Slit-Lamp from Tokyo, Japan) was used to assess pterygium severity. Images at 16 × magnification were captured for subsequent analysis. In evaluating pterygium's relation to cornea, three distinct parameters were recorded. Pterygium's size was determined through thresholding of external photos using ImageJ ((ImageJ, National Institutes of Health,Bethesda, MD). The horizontal length (HL) was measured as distance from nasal limbus to the pterygium's apex. The vertical length (VL) represented distance between two limbal points where pterygium intersected with limbus. The thickness was determined by identifying highest point of anterior corneal surface elevation in the Sirius Scheimpflug Analyzer map.

### The defination of natural-light condition and low-light condition

According to the International Commission on Illumination, the intensity of natural-light condition is defined as 100–130 lx, while scotopic levels of illumination is defined as < 0.05 lx [17]. In this study, natural-light condition was 120.8 lx, and the low-light condition was 0.04 lx (Digital lux meter AS813, Smart Sensor Shenzhen, China).

### Pupil diameter in natural and low ambient illumination

We chose 3 mm as the pupil diameter in natural-light illumination. The scotopic pupil size was measured with Sirius Scheimpflug Analyzer. All eyes were examined by the same examiner in dim illumination. One minute of dark adaptation was allowed before each measurement. The mean and SD of pupil diameters were displayed as a diagram.

### Measurement of corneal astigmatism and aberrations

Sirius Scheimpflug Analyzer quantified corneal astigmatism and HOAs. All eyes were examined by the same examiner. Findings included front and back corneal refractive power at 3 and 7 mm zone, corneal simulated ketatomety (Sim K) parameters. All parameters were expressed in diopters. Astigmatism type was defined as with-the-rule (steep axis between 60—120 degrees); against-the-rule (steep axis < 30 degrees or > 150 degrees); or oblique (steep axis 30—60 degrees or 120—150 degrees). Corneal HOAs and total aberration expressed in root mean square (RMS) for 3 and 7 mm analysis area were obtained.

This study followed the tenets of the Declaration of Helsinki and was approved by Shanghai Aier Eye Ethics Committee. Written informed consent was obtained from all subjects.

### Statistical analysis

All statistical analyses were performed using IBM SPSS version 20 (SPSS Inc., Chicago, IL, USA). Independent samples *t-* test was used to determine if there was a statistically significant difference between two groups for normally distributed continuous variables. If a distribution could not be normalized by logarithmic transformation, the Mann–Whitney U test was used. The correlation between pterygium severity of and corneal parameters of pterygium eyes was evaluated using Spearman correlation test. *P-* values less than 0.05 were considered to be statistically significant.

## Results

The median UCVA (approxETDRS letter scores) of pterygium eyes was 69, with upper and lower quartiles of 65 and 73. The median of UCVA (approxETDRS letter scores) of control eyes was 80, with upper and lower quartiles of 73 and 84. UCVA was significantly lower in pterygium eyes (*P* < 0.001). BCVA was comparable between tgroups (*P* = 0.509) (Table 1).

**Table 1:** Visual acuity (approxETDRS letter scores) in the two groups

	Pterygium eye median (QR)	Control eye median (QR)	*P*^a^
UCVA	69 (65, 73)	80 (73, 84)	< 0.001
BCVA	77 (69, 82)	80 (73, 84)	0.509

^a^Mann–Whitney U test (median( upper QR, lower OR))

The HL of pterygium ranged from 0.33 to 5.06 mm, with a mean of 2.00 ± 1.12 mm. The VL of pterygium ranged from 2.38 to 8.92 mm, with a mean of 5.11 ± 1.17 mm. The pterygium thickness ranged from 36 to 458 μm, with a mean of 192.93 ± 118.42 μm. Pterygium HL (Rho: -0.531 and *P* < 0.001), VL (Rho: -0.322 and *P* = 0.012) and thickness (Rho: -0.399 and *P* = 0.002) were found to be negatively correlated with UCVA (Table 2). There were no statistically correlation between the indices and the BCVA of pterygium (Table 2).

**Table 2:** Corneal refractive power (D) in two groups

	Pterygium eye	Control eye	*P*
Front (3 mm) (D)	43.17 ± 2.68	44.39 ± 1.53	< 0.01^b^
Back (3 mm) (D)	-6.36( -6.55, -6.10)	-6.36( -6.34,-6.21)	0.98^a^
Front (7 mm) (D)	42.07 ± 2.66	44.12 ± 1.39	< 0.01^b^
Back (7 mm) (D)	-5.98( -6.23, -5.35)	-6.23( -6.33, -6.09)	< 0.01^a^
Sim K (D)	43.12( 41.97, 44.29)	44.09( 43.37, 45.27)	< 0.01^a^

*QR* quartile range

^a^Mann–Whitney U test (median (upper QR, lower OR))

^b^Independent samples *t* test ($\overline{{\text{x}} }$ ± s)

In the pterygium and control groups, pupil diameters measured by Sirius Scheimpflug Analyzer under scotopic conditions were 6.88 ± 0.60 mm and 6.87 ± 0.67 mm respectively. No statistically significant difference in pupil diameter was observed between the two groups(*P* = 0.943, independent samples *t*-test). Therefore, for corneal astigmatism and aberration analysis under natural-light and low-light conditions, the corneal zones of 3 mm and 7 mm were selected.

Front corneal refractive power was significantly lower in the pterygium group compared to control group. For back corneal refractive power in the 3 mm zone, no statistically significant difference was observed between the two groups. For the back corneal refractive power in the 7 mm zone, K values were significantly higher in the pterygium group compared to the contol group. Detailed data about the K values of the front and back cornea and Sim K was presented in Table 2.

The astigmatism of the front cornea at both 3 mm and 7 mm zones, back cornea at 7 mm zone and Sim K were significantly higher in the pterygium group compared to the control eyes. For astigmatism of the back cornea at 3 mm zone, no significant difference was observed between the two groups (Table 3). The HL and thickness of the pterygium were positively correlated with all astigmatic values. There was also a relatively weak positive correlation between the VL and astigmatic values except for the astigmatic values of the back cornea at 3 mm zone (Table 4).

**Table 3:** Topographic astigmatism in the two groups

	Pterygium eye	Control eye	*P*^a^
Front corneal astigmatism (3 mm) (D)	1.67 (0.64, 4.36)	0.82 (0.49, 1.28)	0.002
Back corneal astigmatism (3 mm) (D)	0.30 (0.18, 0.63)	0.34 (0.21, 0.45)	0.287
Front corneal astigmatism (7 mm) (D)	3.26 (1.08, 11.08)	0.68 (0.37, 1.13)	< 0.001
Back corneal astigmatism (7 mm) (D)	0.77 (0.22, 2.97)	0.19 (0.12, 0.30)	< 0.001
Sim K astigmatism (D)	1.47 (0.72, 8.70)	0.69 (0.41, 1.18)	< 0.001

^a^Mann–Whitney U test (median (upper QR, lower OR))

**Table 4:** The correlation between pterygium sizes and corneal astigmatism and aberrations

	HL		VL		Thickness	
	r_s_	*P*	r_s_	*P*	r_s_	*P*
Front corneal astigmatism (3 mm)	0.636	< 0.001	0.377	0.003	0.448	< 0.001
Back corneal astigmatism (3 mm)	0.452	< 0.001	0.252	0.052	0.295	0.022
Front corneal astigmatism (7 mm)	0.809	< 0.001	0.556	< 0.001	0.692	< 0.001
Back corneal astigmatism (7 mm)	0.574	< 0.001	0.405	0.001	0.512	< 0.001
Sim K astigmatism (D)	0.705	< 0.001	0.422	0.001	0.566	< 0.001
Total corneal abberation (3 mm)	0.444	< 0.001	0.244	0.06	0.337	0.008
Higher-order abberation (3 mm)	0.515	< 0.001	0.283	0.029	0.354	0.006
Total corneal abberation (7 mm)	0.456	< 0.001	0.101	0.442	0.360	0.005
Higher-order abberation (7 mm)	0.413	< 0.001	0.105	0.425	0.402	0.001

r_s_: Spearman's rank correlation coefficient

**Fig. 1:**
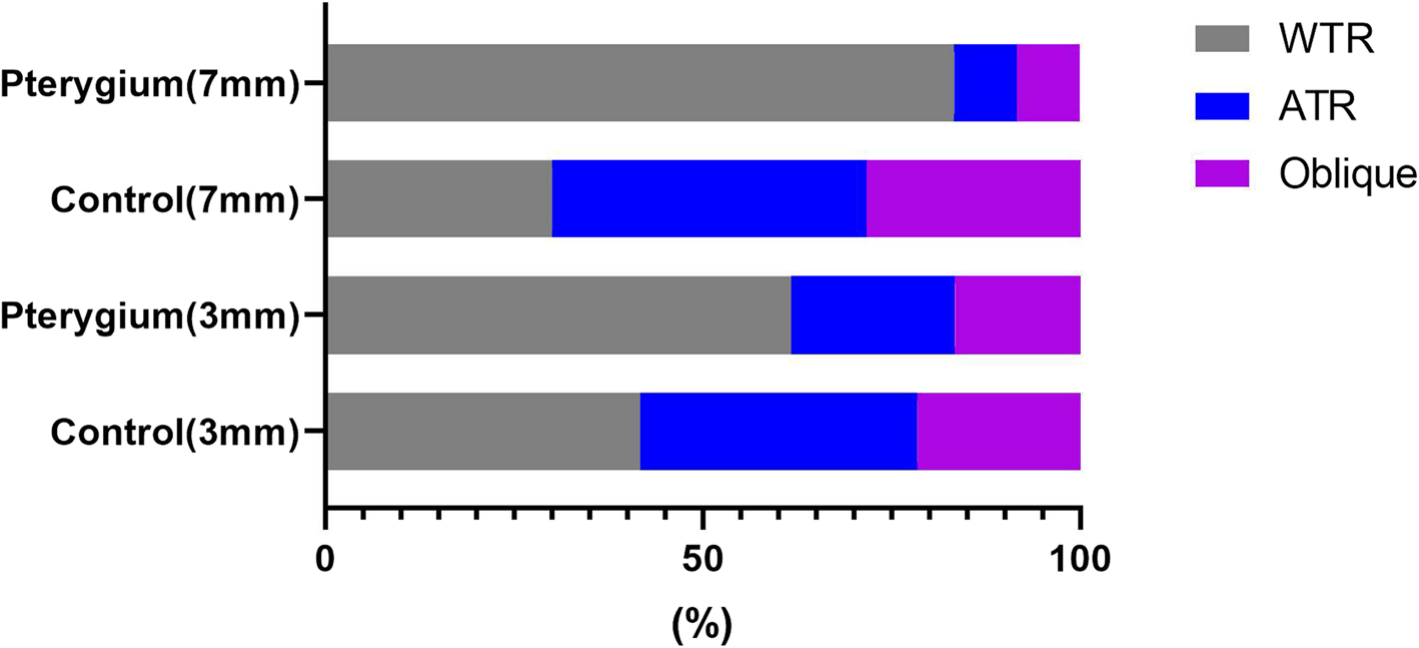
The distribution of types of astigmatism of the front cornea in pterygium and control eyes (WTR: with-the-rule astigmatism, ATR: against-the-rule astigmatism, Oblique: oblique astigmatisam)

Regarding the types of astigmatism of the front cornea, with-the-rule astigmatism,against-the-rule astigmatism and oblique astigmatism were present in 37 eyes (61.7%),12 eyes (21.7%) and 10 eyes (16.7%) in pterygium eyes, and 25 eyes (41.7%), 22 eyes (36.7%) and 13 eyes (21.7%) in control eyes at 3 mm zone (*P* = 0.004, chi-square test). At the 7 mm zone, the 3 types of astigmatism were present in 50 eyes (83.3%), 5 eyes (8.3%) and 5 eyes (8.3%) in pterygium group, and 18 eyes (30%), 25 eyes (41.7%) and 17 eyes (28.3%) in control group (*P* = 0.015, chi-square test) (Fig. 1).

**Fig. 2:**
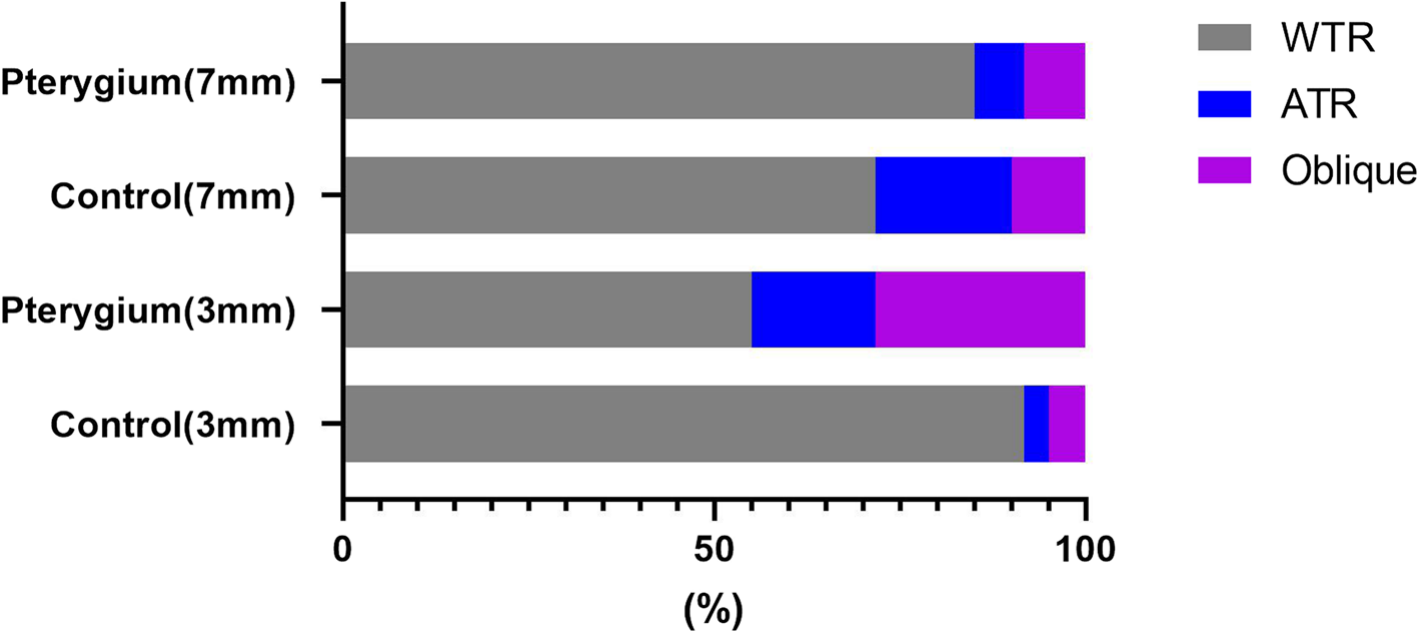
The distribution of types of astigmatism of the back cornea in pterygium and control eyes (WTR:with-the-rule astigmatism, ATR: against-the-rule astigmatism, Oblique: oblique astigmatisam)

The back corneal astigmatism was with-the-rule in 33 eyes (55%), against-the-rule in 10 eyes (16.7%) and oblique in 17 eyes (28.3%) at 3 mm zone in pterygium group and these numbers were 55 eyes (91.7%), 2 eyes (3.3%) and 3 eyes (5%) respectively in the control group (*P* < 0.001, chi-square test). At the 7 mm zone, the 3 types of astigmatism were present in 51 eyes (85.0%), 4 eyes (6.7%), 5 eyes (8.3%) and 43 eyes (71.7%), 11 eyes (18.3%), 6 eyes (10.0%) in the pterygium group and the control group (*P* < 0.001, chi-square test) (Fig. 2).

RMS values were significantly higher in the pterygium group compared to the control group (Table 4) and were positively correlated with both pterygium HL and thickness. There was only a weak positive correlation between pterygium VL and no statistical correlation with HOA at 3 mm zone. Details of the correlation between RMS values of optical aberrations and pterygium HL, VL and thickness on the corneal surface were documented in Table 5.

**Table 5:** The Change of RMS Values in corneal HOAs and total aberrations between pterygium group and control group

Parameters (RMS)	Pterygium eye	Control eye	*P*^a^
Total abberations (3 mm)	0.42 (0.26, 0.82)	0.27 (0.15, 0.40)	0.001
HOAs (3 mm)	0.21 (0.14, 0.40)	0.11 (0.09, 0.19)	< 0.001
Total abberations (7 mm)	3.94 (2.60, 5.61)	2.30 (1.87, 3.16)	< 0.001
HOAs (7 mm)	2.96 (2.04, 5.03)	1.84 (1.54, 2.14)	< 0.001

^a^Mann–Whitney U test (median (upper QR, lower OR))

Comparing parameters of different corneal 3 mm and 7 mm zones, representing the natural-light and low-light conditions, differences in anterior astigmatism, posterior astigmatism, total aberrations and HOAs were significantly larger at 7 mm zone than at 3 mm zone in pterygium versus control groups (*P* = 0.003, *P* < 0.001, *P* = 0.003, *P* < 0.001) (Additional file 1).

## Discussion

Although BSCVA values were comparable between the pterygium and control groups, UCVA values were statistically lower in the pterygium group. Pterygium caused a decrease in corneal power and an increase in corneal astigmatism on the front surface. For the back corneal surface, the corneal power and astigmatism of the pterygium group did not change statistically compared to the control group at 3 mm zone. RMS values of corneal total and HOAs in the pterygium group were higher than those in the control group. The ptergium HL and thickness were moderately to strongly correlated with astigmatism and RMS of aberrations, while pterygium VL showed no or just mild correlation with the corneal indices. The front and back corneal astigmatism, total and HOAs were higher in 7 mm area than in 3 mm area.

Patients with pterygium often presented with visual complaints, such as decreased visual acuity, glare or diplopia [18]. Pterygium induced both refractive and topographic changes, considered contributors to visual impairment [18–21]. Traditionally, however, corneal power and astigmatism have been calculated based on anterior corneal measurements only, assuming a fixed posterior/anterior curvature ratio to estimate the contribution of back corneal power. There would be minimal deviation in normal eyes. In pterygium eyes, the anterior surface of the cornea flattened at the 3 mm zone, while the posterior surface had no obvious variations. The association between the anterior and posterior surface curvature ratio was not a fixed ratio, resulting in error when estimating total corneal astigmatism from only anterior corneal measurements during cataract surgery or similar occasions.

Both front and back corneal astigmatism moderately or strongly correlated with pterygium HL and thickness, while pterygium VL didn’t correlate with the posterior corneal astigmatism at 3 mm zone and mildly or moderately with the other astigmatic parameters. Aberration parameters including total corneal aberrations and HOAs were all higher in the pterygium group than in controls. The RMS values of optical aberrations seemed to moderately or strongly correlated with pterygium HL and moderately with thickness. The VL only mildly correlated with HOAs at 3 mm zone and didn’t correlate with other RMS values. Thus, pterygium HL and thickness affected the visual function while longer VL was not an indication for surgical incision.

In low-light conditions, pupil diameter was 6.88 ± 0.60 mm and 6.87 ± 0.67 mm in pterygium and control groups. We chose the corneal parameters and RMS of aberrations in 7 mm corneal zone to simulate the low-light condition and 3 mm to simulate the natural-light condition. The astigmatism of front and back corneal surface, total aberrations, and HOAs of pterygium eyes were much larger at 7 mm zone than at 3 mm zone. This indicated visual function was worse in low-light condition. The author believed patients with pterygium had poor visual function in low-light conditions. Those who worked in relatively dim environments should be advised to undergo surgery earlier.

## Conclusions

In conclusion, the pterygium impaired visual function by inducing focal corneal flattening, severe astigmatism and increased corneal aberrations, not just by invading the visual axis or distorting the central topography. Pterygium HL and thickness affected visual function more than pterygium width. In low-light conditions, visual function worsened due to increased corneal astigmatism values and aberrations.

### Supplementary Information

**Additional file 1.** The difference of the astigmatism and abberations at 3 mm and 7 mm zone.

## Data Availability

All data supporting the conclusions of this study are included in the present article.
