# Supplementary figures and images for: The effect of pterygium on front and back corneal astigmatism and aberrations in natural-light and low-light conditions
Source: BMC Ophthalmol. 2024 Jan 4;24:7. doi: 10.1186/s12886-023-03270-z (PMC10768295; doi:10.1186/s12886-023-03270-z)

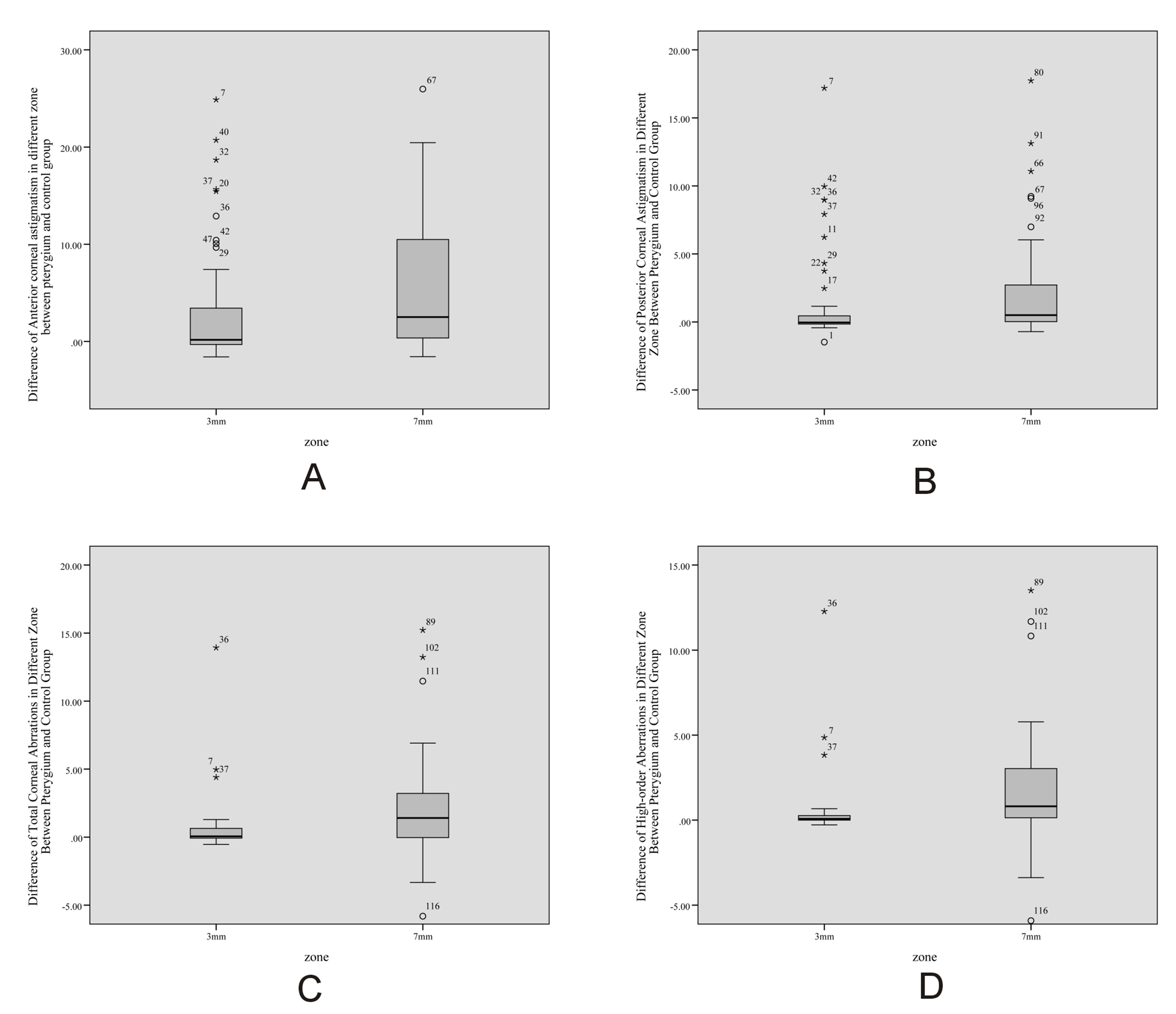

Supplement: Supplementary file 1 — Additional file 1. The difference of the astigmatism and abberations at 3 mm and 7 mm zone. [file 12886_2023_3270_MOESM1_ESM.jpg]
